# Editorial: Inflammation and aging in chronic and degenerative diseases: Current and future therapeutic strategies

**DOI:** 10.3389/fphar.2022.1122786

**Published:** 2023-01-04

**Authors:** Luca Falzone, Saverio Candido, Anca Oana Docea, Daniela Calina

**Affiliations:** ^1^ Epidemiology and Biostatistics Unit, National Cancer Institute IRCCS Fondazione “G. Pascale”, Naples, Italy; ^2^ Department of Biomedical and Biotechnological Sciences, University of Catania, Catania, Italy; ^3^ Department of Toxicology, University of Medicine and Pharmacy of Craiova, Craiova, Romania; ^4^ Department of Clinical Pharmacy, University of Medicine and Pharmacy of Craiova, Craiova, Romania

**Keywords:** inflammation, aging, inflammaging, cancer, neurodegenerative disorders, osteoarthritis, therapeutics

Inflammation and aging represent the most common risk factors for several chronic and degenerative disorders (Furman et al., 2019). In the last decades, it was widely demonstrated how these two pivotal determinants of human pathologies are strongly associated with each other in a dual relationship where aging induces a pro-inflammatory state in the organism and inflammation, in turn, leads to the activation of cellular and molecular pathways involved in cell senescence and aging (Chung et al., 2019; Zhu et al., 2021).

Of note, inflammation and aging are both pathophysiological processes that have been associated with an increased risk of different chronic-degenerative diseases, including tumors, neurological and cardiovascular disorders (Gupta et al., 2018). Due to the strict relationship existing between inflammation and aging, the new term “inflammaging” has been coined to describe a condition characterized by chronic and systemic low-grade inflammation occurring during aging and potentially associated with the alteration of several cellular and molecular pathways and the development of different pathologies (Franceschi et al., 2000).

In the last decades, the main pathogenetic mechanisms driven by both inflammation and aging have been widely described. More in detail, both conditions have been widely associated with the accumulation of genetic and epigenetic alterations responsible for the alteration of cell proliferation and apoptosis responsible for the neoplastic transformation of cells and the development of tumors (Candido et al., 2021; Zhu et al., 2021). Similarly, cell senescence due to aging has been widely associated with the impairment of mitochondrial as well as proteosome and lysosome functions responsible for the accumulation of aberrant or misfolded proteins often observed in different neurodegenerative disorders (Sikora et al., 2021). Besides these well-known pathogenetic mechanisms related to inflammaging, other processes are involved in age-related and inflammatory-related diseases including enzyme dysfunctions, cell death, impaired tissue renewal and tissue degeneration (Li, 2013).

Despite the long-term effects of inflammation and aging are well established, there is still a debate on potential therapeutic interventions aimed at limiting both processes. If anti-inflammatory therapeutic interventions are currently available, antiaging strategies are still ineffective and mainly based on the adoption of antioxidant and cytoprotective agents (Flatt et al., 2013).

Therefore, it is evident how a better knowledge of inflammaging and novel therapeutic strategies aimed at reducing inflammation during aging are mandatory to effectively manage age- and inflammatory-related diseases.

On these bases, the aim of the Research Topic entitled “Inflammation and Aging in Chronic and Degenerative Diseases: Current and Future Therapeutic Strategies” was to collect the latest update on the molecular and cellular determinants responsible for inflammatory processes during aging as well as the role of aging in the onset of chronic-degenerative diseases. Particular attention was paid to approved and under-developing therapeutic strategies for the treatment of pathologies associated with inflammaging as well as to the epigenetic factors associated with these processes.

Overall, a total of 14 tentative papers were submitted to the Research Topic, of which seven were published and are gaining great interest in the scientific community. More in detail, one review article and six original research articles were accepted for publication.

Noteworthy, the majority of the papers published within the Research Topic (four out of seven) were interested in unveiling the pathogenesis of osteoarthritis and in evaluating the therapeutic potential of novel drugs for the treatment of this pathology suggesting how osteoarthritis represents a severe threat in public health and the need for novel and effective therapeutic strategies.

Specifically, Zeng et al. published a comprehensive review of the literature on the role of HIF-1α in the regulation of autophagy and apoptosis of chondrocytes as well as in the decrease of inflammatory cytokines and in chondrocyte survival. They also elegantly described the role of HIF-1α as a crucial therapeutic target for the protection of chondrocytes and the prevention of osteoarthritis.

As regards novel therapeutic interventions in osteoarthritis, Xian et al. evaluated the protective effects of the natural molecule Evodiamine (EV) extracted from *Evodia rutaecarpa* in osteoarthritis through *in vitro* and *in vivo* experiments. The authors demonstrated that EV is effective in reducing the levels of pro-inflammatory cytokines, including NO, IL-6, TNF-α, and PGE2, while EV is effective in reducing cartilage degeneration *in vivo*. These data suggest how EV could reduce the risk of cartilage degradation by modulating several inflammatory pathways like the NF-κB signaling pathway.

Similarly, the group of Lin et al. evaluated the protective effects of another phytochemical alkaloid extracted from natural herbs, Nitidine Chloride (NitC), which exerts anti-inflammatory and anti-oxidative action in osteoarthritis. More in detail, the authors treated IL-1β-induced osteoarthritis *in vitro* models with NitC demonstrating the inhibition of COX2, iNOS, MAPK, and NF-κB pathways responsible for the degradation of cartilage.

Another original research by Zheng et al., tested the efficacy of the MAPK/MEK selective inhibitor selumetinib in osteoarthritis. Through *in vitro* and *in vivo* experiments, the authors demonstrated that selumetinib reduces the degradation of extracellular matrix and induces the synthesis of cartilage by inhibiting the RIP1/RIP3/MLKL and the RANKL-induced NF-κB and MAPK pathways in chondrocytes. The data obtained strongly supported the adoption of selumetinib to protect chondrocytes from degradation and improve the activity of both chondrocytes and osteoclasts.

Besides these novel therapeutic interventions for the treatment of osteoarthritis, also the mechanisms and therapeutic interventions for inflammatory- and aging-mediated neurodegeneration were investigated. In particular, Wei et al. investigated the role of α7 nicotinic acetylcholine receptor (α7nAChR) as a novel therapeutic target in aging-related neurocognitive disorders. In detail, the authors demonstrated that α7nAChR agonist reduces the levels of IL-1β and activate the BDNF pathway after surgery alleviating neurodegeneration and exerting neuroprotective effects suggesting how α7nAChR may represent a novel therapeutic strategy for the treatment of surgery-related neurodegeneration.

Similarly, Zhou et al. unveiled the protective role of glutamine as a regulator of mTOR signaling and autophagy. The authors demonstrated that chronic glutamine deprivation induces senescence through the activation of the Akt-mTOR pathway and the impairment of lysosome functions suggesting how glutamine supplementation in the elderly can be used as a nutritional therapy to reduce the detrimental effects of aging.

Finally, Fang et al., investigated the neuroprotective effects of the anticancer drug RRx-001 in cellular and *in vivo* models of LPS-induced neuroinflammation. In particular, they demonstrated that RRx-001 suppresses the activation of microglia and the production of pro-inflammatory cytokines by inhibiting TAK1, proposing RRx-001 as a candidate drug for the treatment of neuroinflammation-related brain diseases.

Overall, the papers in this Research Topic unveiled some novel therapeutic strategies for the treatment of inflammatory- and aging-related disorders, however, further studies are needed to effectively validate the therapeutic potential of natural extracts or selective inhibitors as modulators of inflammaging and effective treatments for neurodegenerative and chronic disorders.
